# Co-occurrence of tuberculosis and diabetes mellitus, and associated risk factors, in Ethiopia: a systematic review and meta-analysis

**DOI:** 10.1016/j.ijregi.2021.10.004

**Published:** 2021-10-20

**Authors:** Ayinalem Alemu, Zebenay Workneh Bitew, Getu Diriba, Balako Gumi

**Affiliations:** 1 Ethiopian Public Health Institute, Addis Ababa, Ethiopia; 2 Aklilu Lemma Institute of Pathobiology, Addis Ababa University, Addis Ababa, Ethiopia; 3 St Paul's Hospital Millennium Medical College, Addis Ababa, Ethiopia

**Keywords:** tuberculosis, diabetes, Ethiopia, co-occurrence, risk factors, BMI: body mass index, DM: diabetes mellitus, EPTB: extrapulmonary tuberculosis, HIV: human immunodeficiency virus, IFG: impaired fasting glucose, MDR-TB: multidrug-resistant tuberculosis, PTB: pulmonary tuberculosis, PRISMA: Preferred Reporting Items for Systematic Reviews and Meta-analyses, OR: odds ratio, TB: tuberculosis, WHO: World Health Organization

## Abstract

**Objective:**

The aim of our study was to estimate the pooled prevalence of tuberculosis (TB) and diabetes mellitus (DM) co-occurrences, and associated risk factors, in Ethiopia.

**Methods:**

In total, 392 articles were identified, and 14 were included in the analysis. The study's quality was assessed using the JBI tool. Data were analyzed using STATA version 16. The pooled prevalences of TB among DM patients and of DM among TB patients, with 95% CI, were estimated. In addition, the pooled OR values for each risk factor were estimated. The study's heterogeneity was assessed using forest plots and the *I*^2^ heterogeneity test. Publication bias was assessed using funnel plots and Egger's regression test.

**Results:**

The pooled prevalence of TB among DM patients was 4.14% (95% CI 2.45–5.83%). The pooled prevalence of DM among TB patients was 12.77% (95% CI 6.91–18.62%). Type 1 DM patients had a higher TB risk (OR 2.70, 95% CI 1.41–3.99). Older age (OR 2.25, 95% CI 1.38–3.13) and family DM history (OR 3.65, 95% CI 1.89–5.41) were associated with DM co-occurrence among TB patients.

**Conclusions:**

Higher prevalences of TB among DM patients and of DM among TB patients were noted in Ethiopia, when compared with the general population. Thus, active screening of DM patients for TB and vice versa is recommended.

## Highlights

• DM patients have a high TB risk, and TB patients can develop DM during their treatment.
• Around 4.14% of DM patients in Ethiopia have TB.
• Around 12.7% of TB patients in Ethiopia develop DM.
• Type 1 DM patients have a higher TB risk.
• Old age and a family history of DM are risk factors for DM in TB patients.

## Introduction

Even though one quarter of the global population is estimated to have been infected with Mtb, only 5–10% have a lifetime risk of falling ill with tuberculosis (TB). The risk of TB is higher among immune-compromised individuals and those with certain underlying diseases (Walker et al., 2013). Those with compromised immune systems, such as people living with HIV, malnutrition, or diabetes (DM), or smokers, have a higher risk of falling ill with TB (WHO, 2020). Many new cases of TB are attributable to five risk factors: undernutrition, HIV infection, alcohol use, smoking, and DM (WHO, 2020). In 2019, an estimated 2.2, 0.76, 0.72, 0.70, and 0.35 million TB cases were attributable to undernutrition, HIV infection, alcohol use disorders, smoking, and DM, respectively (WHO, 2020).

Aside from the traditional risk factors, DM is increasingly being recognized as an independent risk factor for TB, and the two often coexist (Yorke et al., 2017). The interaction between DM and TB is a major public health concern because of the rapidly rising levels of DM. DM increases the risk of TB infection by two to three times (Harries et al., 2013). Immune mechanisms contributing to the increased susceptibility of DM patients to TB are based on defects in bacterial recognition, phagocytic activity, and cellular activation that results in impaired production of chemokines and cytokines (Ayelign et al., 2019). The relationship between TB and DM is bidirectional, whereby TB patients also develop new DM cases during their treatment (Niazi & Kalra, 2012)Niazi & Kalra, 2012). The World Health Organization (WHO) recommends three intervention strategies: establishing mechanisms of collaboration between TB and DM control programs; early detection and management of TB in patients with DM; and early detection and management of DM in TB patients (WHO, 2015).

TB is a major public health problem in Ethiopia, with an incidence of 140/100 000 population. Ethiopia is included under high TB-, TB/HIV-, and MDR-TB-burden countries across the globe (WHO, 2020). In 2018, 114 233 TB cases were notified in the country, with a case/fatality ratio of 17% (9–25%) (WHO, 2019). Moreover, the burden of non-communicable diseases, including DM, is increasing in Ethiopia, with 3.2% in adults (IDF, 2019), ranging from 2.0% to 6.5% (Bishua et al., 2019).

Diabetic patients are susceptible to TB. Individual studies conducted in Ethiopia have also confirmed this. A TB prevalence among DM patients of more than 5.0% has been reported in Ethiopia (Gedfew et al., 2020; Abera & Ameya, 2018). Studies have also reported that DM is a common phenomenon among TB patients in Ethiopia, with a prevalence of up to 15.8% (Damtew et al., 2014). However, the findings of individual studies are inconclusive, and there is a scarcity of updated data on the status of TB and DM co-occurrences in the country. Thus, our study aimed to assess the burden of TB and DM co-occurrences, and associated risk factors, in Ethiopia.

## Methods

### Search strategy

Systematic article searching was conducted using electronic databases (PubMed, CINAHL, DOAJ, African Index Medicus) and other gray literature sources (Google, Google Scholar, WorldCat). This was performed independently by two authors (AA and GD) under the consultation of a senior librarian at the Ethiopian Public Health Institute. Inconsistencies were resolved by a third author (ZWB). The keywords used for searching included tuberculosis, diabetes, co-occurrences, risk factors, associated factors, determinants, predictors, and Ethiopia. These keywords were used in conjunction with the Boolean operators AND and OR. The search string for the PubMed database was ((((”Tuberculosis” [Mesh]) OR (TB)) AND (“Diabetes Mellitus” [Mesh] OR “Diabetes Mellitus, Type 1” [Mesh])) OR (DM)) AND (“Ethiopia” [Mesh]) (Additional file 1).

### Study selection procedure

**Figure 1:**
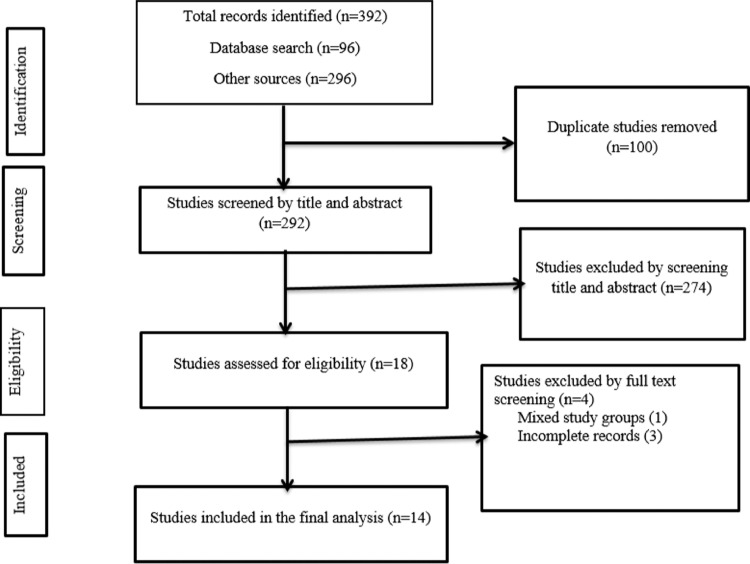
Flowchart describing the selection of studies for the systematic review and meta-analysis of TB and DM co-occurrences in Ethiopia

This study was conducted following the Preferred Reporting Items for Systematic Reviews and Meta-Analyses (PRISMA) reporting checklist (Hutton et al., 2015; Knobloch et al., 2011) (Additional file 2). A stepwise approach was followed to select the eligible articles included in the final analysis. The article selection procedure was conducted independently by two authors (AA and GD) using predefined inclusion criteria, and the inconsistencies were resolved by a third author (ZWB). Initially, all the identified articles (*n* = 392) were exported to the EndNote X8 citation manager, and 100 duplicates were removed. Next, 292 articles were screened by title and abstract. Around 18 articles that passed the first stage were assessed through a full-text review. During this review, the study subjects, study design, study quality, and outcome were considered. Finally, 14 articles became eligible for data extraction. During article eligibility assessment, the PICOS (Population, Intervention, Comparison, Outcome, Study design, Study setting) criteria were assessed (Figure 1).

### PICOS criteria

Participants: TB/DM patients
Intervention: Not applicable
Comparator: DM patients without TB/TB patients without DM
Outcome: TB among DM patients/DM among TB patients
Study design: Observational studies
Study setting: Any setting across Ethiopia

### Inclusion and exclusion criteria

Articles that reported TB prevalence among DM patients, or DM co-occurrence among TB patients, or articles that reported associated factors for TB and DM co-occurrences in Ethiopia, and that were published in the English language were included. Articles without full text, or that did not separately report the prevalence for each group, or that were not objectively designed to assess TB prevalence among DM patients or DM prevalence among TB patients were excluded.

### Data extraction

Data were extracted independently by two authors (AA and GD), and the inconsistencies were resolved by a third author (ZWB). The extracted data included study characteristics, such as author, publication year, regional state, study setting, data collection period, study participants’ age range, sample size, number of DM patients with TB, and number of TB patients with DM. Demographic and behavioral data, and clinical factors for TB and DM co-occurrences, were also extracted. The data were summarized using Microsoft Excel 2016 spreadsheets (Tables 1 and 2).

**Table 1:** Characteristics of the individual studies on tuberculosis infection among diabetes patients in Ethiopia included in the current systematic review and meta-analysis

Authors, year	Study region	Study setting	Study period	Study design	Study age group	DM patients			DM type			
						Total number	TB infected	Type of TB	Type 1		Type 2	
									Number	TB infected	Number	TB infected
Abera & Ameya, 2018	SNNP	Hawassa Adare Hospital	Mar to May, 2015	Cross-sectional	17–95	207	11	All PTB	–	–	–	–
Amare et al., 2013	Amhara	Dessie Referral Hospital	Feb to Apr, 2012	Cross-sectional	12–82	225	14	All PTB	40	3	185	11
Andualem & Malede, 2021	Amhara	Debre Tabor General Hospital	Mar to May, 2019	Cross-sectional	16–71	258	7	All PTB	–	–	–	–
Feleke et al., 1999	Addis Ababa	Tikur Anbessa Specialized Teaching Hospital	Sep 1989 to 1996	Cross-sectional	–	1352	78	PTB = 56 EPTB = 8 Disseminated TB = 7	619	54	733	17
Gedfew et al., 2020	Amhara	Debre Markos Referral Hospital	Jan 2013 to Dec 2017	Retrospective cohort	18–79	433	26	PTB = 20 EPTB = 6	224	19	209	7
Jerene et al., 2017	Amhara and Oromia	Bishoftu, Shashemene, Debrebirhan, Debretabor Hospitals	Feb to June, 2015	Cross-sectional	–	888	6	–	–	–	–	–
Tiroro et al., 2015	Addis Ababa	Tikur Anbessa Specialized Teaching Hospital	Jan 2010 to Jan 2014	Cross-sectional	18–88	681	26	PTB = 24 EPTB = 2	121	10	551	16

SNNP: Southern Nations Nationalities and Peoples, DM: diabetes mellitus, TB: tuberculosis, PTB: pulmonary tuberculosis, EPTB: extrapulmonary tuberculosis

**Table 2:** Characteristics of individual studies on diabetes mellitus among tuberculosis patients in Ethiopia included in the current systematic review and meta-analysis

Author, year	Study region	Place/setting	Study period	Study design	Study age group	TB patients			Type of TB			
						Total number	Had DM	Had IFG	PTB		EPTB	
									Number	Had DM	Number	Had DM
Ashebir, 2015	Addis Ababa	St Paul Millennium Medical College	June 2014 to Feb 2015	Cross-sectional	-	205	17	53	205	17	–	–
Damtew et al., 2014	Addis Ababa	St. Peter Specialized Hospital	Feb to May 2014.	Cross-sectional	15–86	120	19	32	120	19	–	–
Getachew et al., 2013	Amhara	Gondar University Hospital	Oct 2011 to Nov 2012	Cross-sectional	14–80	199	17	59	199	17	–	–
Gezahegn et al., 2020	Oromia	Bale Zone Health Institutions	Mar 30 to Apr 30, 2019	Cross-sectional	All	316	16	–	233	7	83	9
Jerene et al., 2017	Amhara and Oromia	Bishoftu, Shashemene, Debrebirhan, Debretabor hospitals	Feb to June 2015	Cross-sectional	–	439	141	–	–	–	–	–
Tenaye et al., 2019	Dire Dawa	25 public and private health facilities in Dire Dawa	Mar 10 to Apr 15, 2017	Cross-sectional	≥18	421	57	125	338	48	83	9
Workneh et al., 2016	Amhara	Health facilities in South-Eastern Amhara	Sep 2013 to Sep 2014	Cross-sectional	15–89	1314	109	139	770	70	544	39
Tulu et al., 2021	Amhara	Felege Hiwot and Debre Tabor Hospitals	Feb 1 to Jun 30, 2017	Cross-sectional	18–62	269	31	67	104	14	165	17

DM: diabetes mellitus, TB: tuberculosis, PTB: pulmonary tuberculosis, EPTB: extrapulmonary tuberculosis

### Risk of bias (quality) assessment of studies

The quality of individual studies was assessed using the Joanna Briggs Institute (JBI) critical appraisal checklist for prevalence studies and cohort studies (Moola et al., 2020). The questions in the checklist were equally scored and then totalled to give a final score out of 100%. The quality score was graded as low if < 60%, medium if 60–80% and high if > 80% (Porritt et al., 2014; Munn et al., 2019) (Additional File 3). Two authors (AA and GD) independently assessed the quality of the studies and the inconsistencies were resolved by a third author (ZWB). The symmetry of the funnel plots was assessed visually, and Egger's regression test was performed to assess publication bias.

### Outcomes

The presence of TB and DM co-occurrences was the primary outcome. This was assessed by estimating the pooled prevalence of TB among DM patients and the pooled prevalence of DM among TB patients. The associated factors contributing to the presence of TB and DM co-occurrences formed the secondary outcome. The pooled odds ratio for each factor was estimated.

### Operational definitions

For this study, diabetes was operationalized as having a fasting blood glucose level of 126 mg/dl or more, while prediabetes/impaired fasting blood glucose was operationalized as having a fasting blood glucose level of 100–125 mg/dl. Older age was taken as the higher age group in the primary studies; in the majority of the studies this was over 45 years.

### Data synthesis and statistical analysis

The data summarized in Microsoft Excel 2016 were exported to STATA version 2016 for statistical analysis. The pooled proportion of DM patients infected with TB and the pooled proportion of TB patients who had DM were estimated, along with 95% CIs. To assess the associated factors for TB and DM co-occurrences, the pooled ORs along with 95% CIs were estimated. The pooled estimates were presented as forest plots. The forest plots and *I*^2^ heterogeneity tests were examined to assess heterogeneity among the studies. *I*^2^ values of 25%, 50%, and 75% were interpreted as the presence of the low, medium, and high heterogeneity, respectively (Sterne & Egger, 2001; Riley et al., 2011). In addition, the presence of publication bias was assessed through visual inspection of the funnel plots and Egger's regression test. Asymmetry of the funnel plots and statistical significance of Egger's regression test (*p* < 0.05) were considered to represent the presence of publication bias.

## Results

### Study characteristics of included studies

After systematic searching, 14 studies (Gedfew et al., 2020; Abera & Ameya, 2018; Damtew et al., 2014; Amare et al., 2013; Andualem & Malede, 2021; Feleke et al., 1999; Jerene et al., 2017; Tiroro et al., 2015; Ashebir, 2018; Getachew et al., 2014; Gezahegn et al., 2020; Tenaye et al., 2019; Workneh et al., 2016; Tulu et al., 2021) were included in the final analysis (Figure 1). Six studies (Gedfew et al., 2020; Abera & Ameya, 2018; Amare et al., 2013; Andualem & Malede, 2021; Feleke et al., 1999; Tiroro et al., 2015) reported on the prevalence of TB among DM patients, while the other seven studies (Damtew et al., 2014; Ashebir, 2018; Getachew et al., 2014; Gezahegn et al., 2020; Tenaye et al., 2019; Workneh et al., 2016; Tulu et al., 2021) were conducted to determine the prevalence of DM among TB patients. The one remaining study (Jerene et al., 2017) assessed both TB prevalence among DM patients and DM prevalence among TB patients. The studies that determined the prevalence of TB among DM patients were reported from Amhara (four studies), Addis Ababa (two studies), Oromia (one study), and SNNP (one study) regions. All of these studies were hospital-based. The study period for these studies ranged from 1989 (Feleke et al., 1999) to 2019 (Andualem & Malede, 2021). Six out of seven studies were conducted after January 2010 (Gedfew et al., 2020; Abera & Ameya, 2018; Damtew et al., 2014, Amare et al., 2013; Andualem & Malede, 2021; Jerene et al., 2017; Tiroro et al., 2015). Most (6/7) of the studies were cross-sectional (Abera & Ameya, 2018; Damtew et al., 2014, Amare et al., 2013; Andualem & Malede, 2021; Feleke et al., 1999; Jerene et al., 2017; Tiroro et al., 2015), while the other used a retrospective cohort study design (Gedfew et al., 2020). The age of DM patients ranged from 12 years (Amare et al., 2013) to 95 years (Abera & Ameya, 2018). The studies that assessed DM co-occurrence among TB patients were reported from Amhara (four studies), Oromia (two studies), Addis Ababa (two studies), and Dire Dawa (one study) regions. All these studies were conducted in healthcare facilities. The study period for these studies ranged from October 2011 (Getachew et al., 2104) to April 2019 (Gezahegn et al., 2020), and all the studies were cross-sectional (Tables 1 and 2).

### Tuberculosis among diabetes mellitus patients

**Figure 2:**
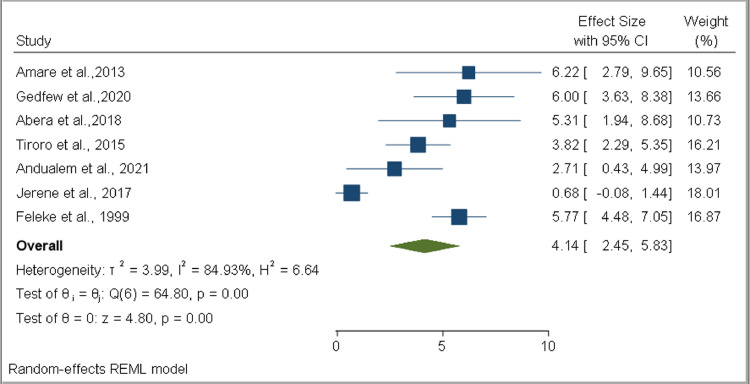
Forest plot for the pooled prevalence of tuberculosis in patients with diabetes mellitus in Ethiopia

**Figure 3:**
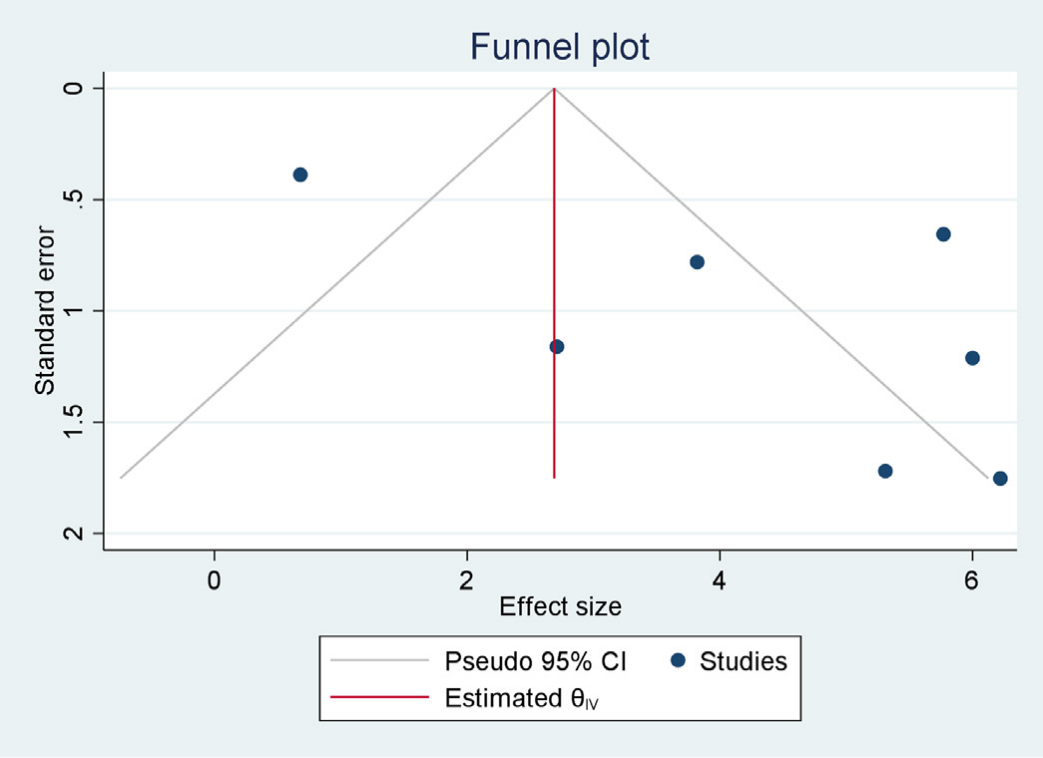
Funnel plot for the pooled prevalence of tuberculosis in patients with diabetes mellitus in Ethiopia

Estimates of the pooled prevalence of TB among DM patients were based on seven studies. The sample size ranged from 207 (Abera & Ameya, 2018) to 1352 (Feleke et al., 1999). Three studies (Abera & Ameya, 2018; Amare et al., 2013; Andualem & Malede, 2021) assessed the prevalence of pulmonary TB (PTB), while the other three studies assessed both PTB and extrapulmonary TB (EPTB) (Gedfew et al., 2020; Feleke et al., 1999; Tiroro et al., 2015). However, one study did not specify whether its was assessing PTB, EPTB, or both types (Jerene et al., 2017). Four studies reported TB prevalence based on DM type (Gedfew et al., 2020; Amare et al., 2013; Feleke et al., 1999; Tiroro et al., 2015). The highest prevalence was reported as 6.2% (Amare et al., 2013), while the smallest reported prevalence was 0.68% (Jerene et al., 2017). However, four of the seven studies reported prevalences of ober 5% (Gedfew et al., 2020; Abera & Ameya, 2018; Amare et al., 2013; Feleke et al., 1999). Estimates of pooled prevalences of TB were based on data collected from 4044 DM patients, of whom 168 developed TB. Based on the random effect model, the pooled prevalence of TB among DM patients was estimated as 4.14% (95% CI 2.45–5.83%, *I*^2^ = 84.93%) (Figure 2). Publication bias was not detected (*p* = 0.1090) (Figure 3). The pooled prevalences of TB among type 1 DM and type 2 DM patients were estimated as 8.56% (95% CI 6.74–10.38%, *I*^2^ = 0.00%) and 2.80% (95% CI 1.93–3.66%, *I*^2^ = 0.00%), respectively. Publication bias was not detected in either group (*p* = 0.76687 and p = 0.0836, respectively). The pooled prevalences of PTB and EPTB among DM patients, based on the available articles that specifically reported the site of TB, were estimated as 4.05% (95% CI 3.31–4.78%, *I*^2^ = 0.00%) and 0.53% (95% CI 0.19–0.88%, *I*^2^ = 0.00%), respectively. Publication bias was not detected in either group (*p* = 0.3895 and *p* = 0.2453, respectively) (Supplementary figure 1).

### Prevalence of DM among TB patients

**Figure 4:**
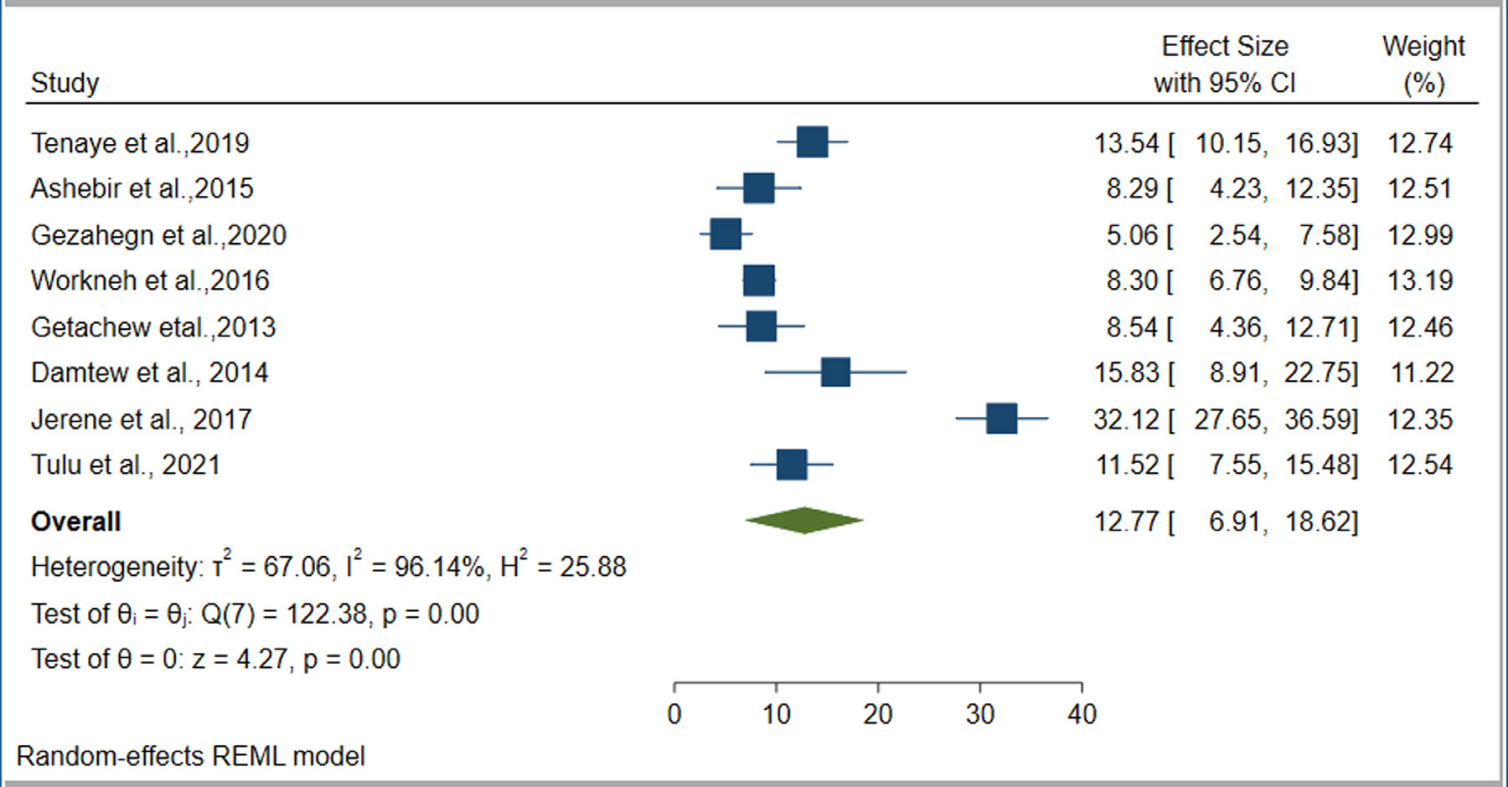
Forest plot for the pooled prevalence of diabetes mellitus co-occurrence among tuberculosis patients in Ethiopia

**Figure 5:**
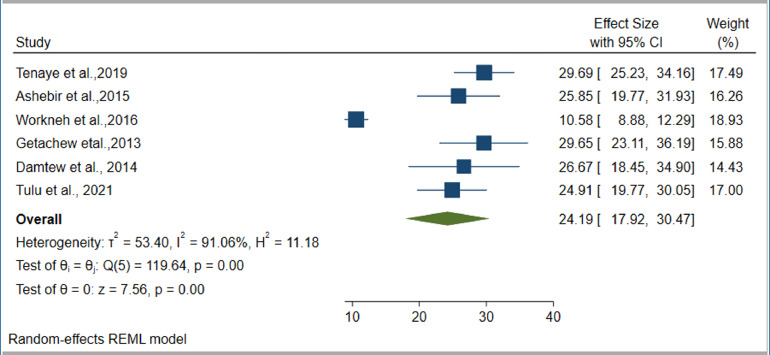
Forest plot for the pooled prevalence of impaired fasting glucose among tuberculosis patients in Ethiopia

**Figure 6:**
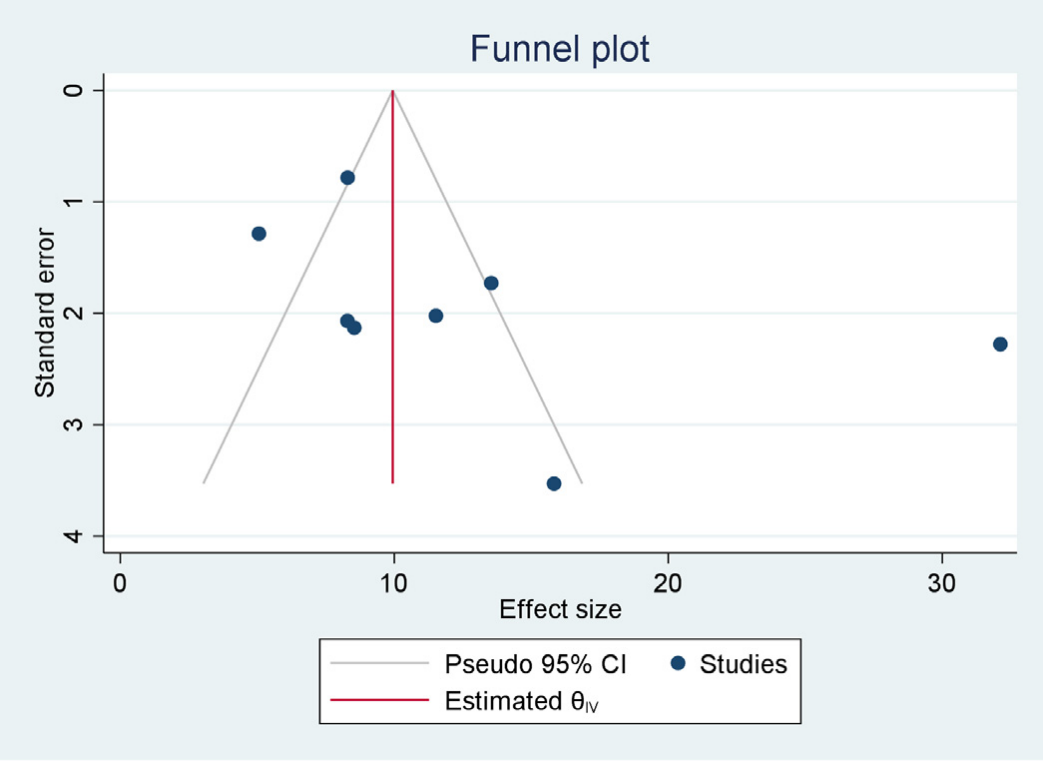
Funnel plot for the pooled prevalence of diabetes mellitus co-occurrence among tuberculosis patients in Ethiopia

**Figure 7:**
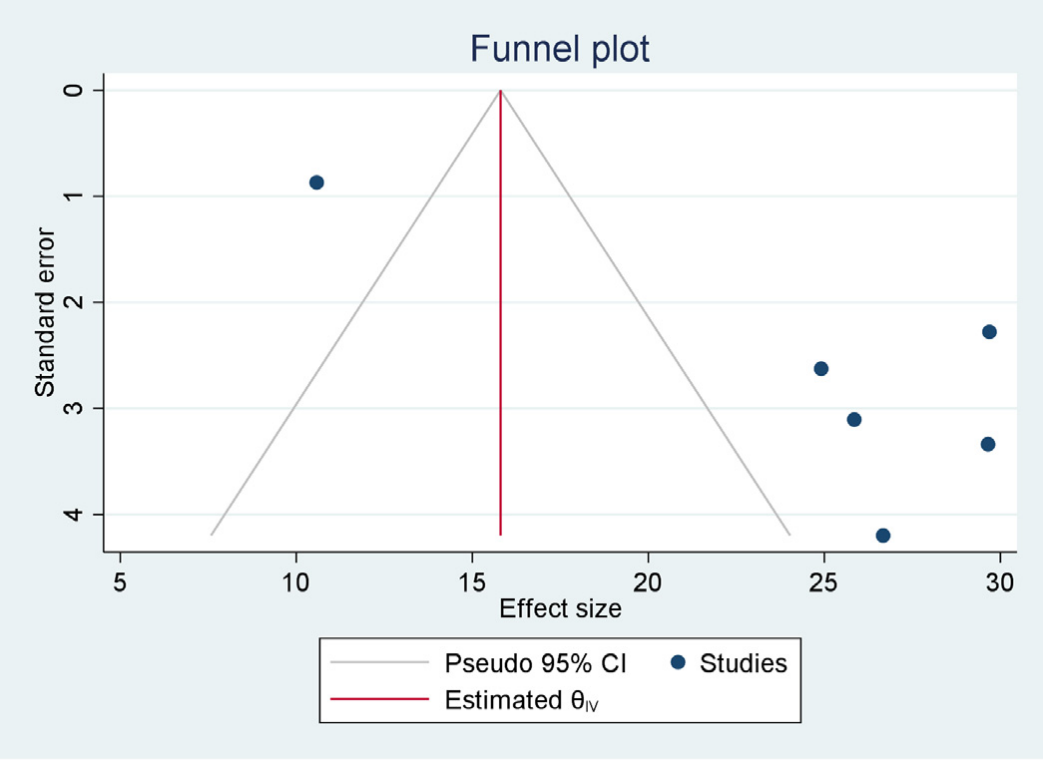
Funnel plot for the pooled prevalence of impaired fasting glucose among tuberculosis patients in Ethiopia

Our estimate of the pooled prevalence of DM co-occurrence among TB patients was based on eight individual studies. The sample sizes ranged from 120 (Damtew et al., 2014) to 1314 (Workneh et al., 2016). Six studies (Damtew et al., 2014; Ashebir, 2018; Getachew et al., 2014; Tenaye et al., 2019; Workneh et al., 2016; Tulu et al., 2021) reported the prevalence of IFG among TB patients. The prevalence of DM per type of TB was reported by four studies (Gezahegn et al., 2020; Tenaye et al., 2019; Workneh et al., 2016; Tulu et al., 2021). The highest prevalence of DM was reported as 32.4% (Jerene et al., 2017), while the smallest reported prevalence was 5.1% (Gezahegn et al., 2020). However, seven out of eight studies (Damtew et al., 2014; Jerene et al., 2017; Ashebir, 2018; Getachew et al., 2014; Tenaye et al., 2019; Workneh et al., 2016; Tulu et al., 2021) reported DM prevalences of over 8%. The pooled prevalence of DM among TB patients was estimated based on data collected from 3293 TB patients, of whom 407 had DM. The pooled prevalence of IFG among TB patients was estimated by data collected from 2528 TB patients, of whom 475 had IFG. Based on the random effect model, the pooled prevalence of DM and IFG among TB patients was estimated as 12.77% (95% CI 6.91–18.62%, *I*^2^ = 96.14%) (Figure 4) and 24.19% (95% CI 17.92–30.41%, *I*^2^ = 91.06%), respectively (Figure 5). Publication bias was not detected for DM (*p* = 0.2440) (Figure 6), but was detected for IFG (*p* = 0.0110) (Figure 7). Specific to the site of TB, the pooled prevalences of DM among PTB and EPTB patients were estimated as 9.79% (95% CI 6.49–13.09%, *I*^2^ = 81.88%) and 9.92% (95% CI 8.01–11.82%, *I*^2^ = 0.00%), respectively. Publication bias was detected among the PTB cases (*p* = 0.0456) but not among the EPTB cases (*p* = 0.6554) (Supplementary figure 1).

### Risk factors for tuberculosis and diabetes mellitus co-occurrences

The associated risk factors for developing TB among DM patients and DM co-occurrence among TB patients were estimated using the available studies conducted so far in Ethiopia. The pooled OR was estimated for the 13 variables associated with developing TB among DM patients, while the pooled OR was estimated for the 12 variables associated with DM co-occurrence among TB patients. These variables were chosen based on their appearance in the primary studies. The variables included socio-demographic, behavioral, and clinical characteristics.

**Figure 8:**
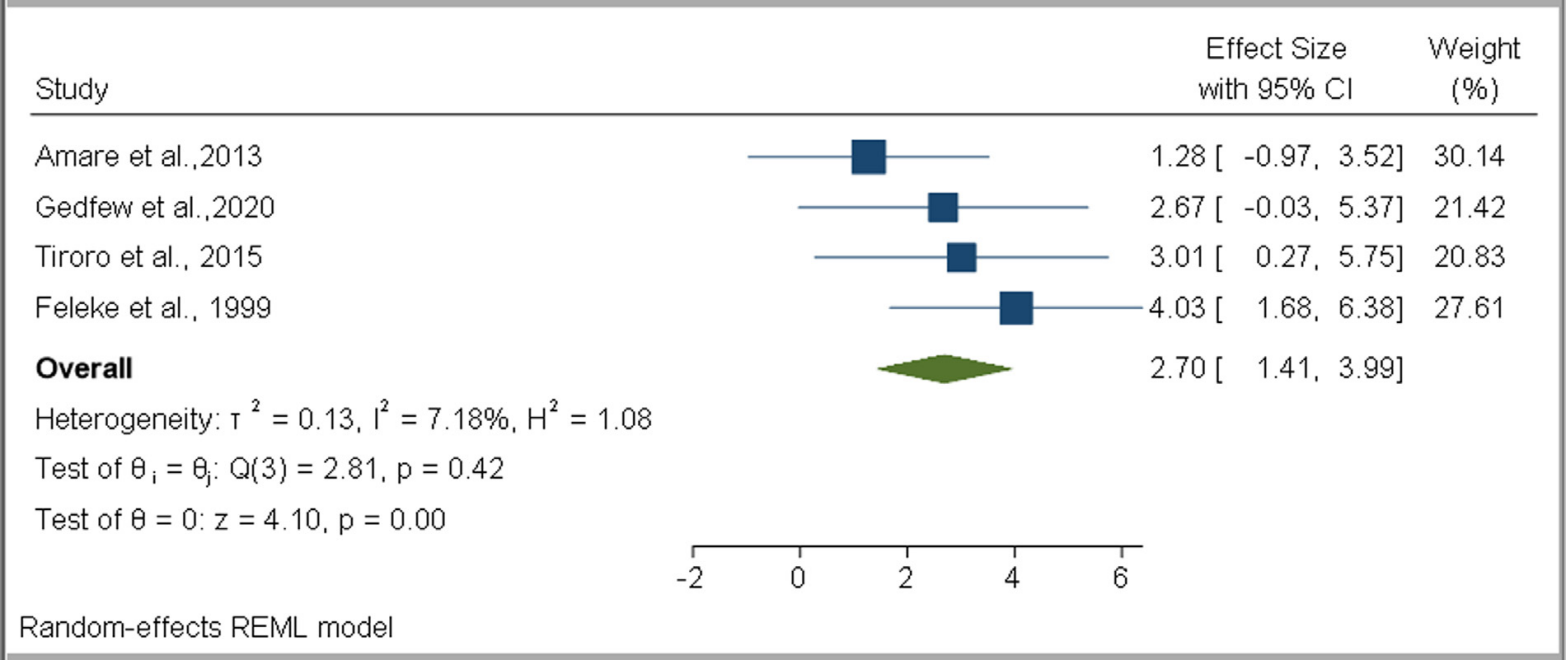
Forest plot for the association of type 1 DM with TB infection among diabetes mellitus patients in Ethiopia

Based on the pooled estimates, only the type of DM was associated with developing TB among DM patients (OR 2.70, 95% CI 1.41–3.99, *I*^2^ = 7.18%) (Figure 8). No statistically significant association was found for male sex (OR 1.38, 95% CI 0.56–2.20, *I*^2^ = 0.00%), urban residence (OR 1.01, 95% CI 0.22–1.81, *I*^2^ = 0.00%), smoking (OR 5.63, 95% CI −2.79 to 14.06, *I*^2^ = 2.08%), alcohol consumption (OR 6.95, 95% CI 0.79–13.10, *I*^2^ = 0.00%), HIV (OR 1.42, 95% CI −0.19–3.03, *I*^2^ = 0.00%), previous TB history (OR 10.64, 95% CI −1.28 to 22.56, *I*^2^ = 8.56%), DM duration more than 10 years (OR 7.21, 95% CI −1.35 to 15.76, *I*^2^ = 0.00%), BMI < 18.5 kg/m^2^ (OR 2.57, 95% CI −1.18 to 6.32, *I*^2^ = 43.09%), family DM history (OR 1.42, 95% CI –0.19 to 3.03, *I*^2^ = 0.00%), close contact with a known TB patient (OR 5.73, 95% CI 0.32–11.13, *I*^2^ = 0.00%), insulin medication (OR 2.11, 95% CI 0.71–3.52, *I*^2^ = 0.00%), and poor glycemic control (OR 1.51, 95% CI 0.37–2.66, *I*^2^ = 0.00%) (Table 3 and Supplementary figure 2).

**Table 3:** Summary of pooled estimates of OR values for associated factors for TB infection among DM patients in Ethiopia

Variable	Odds ratio			
	Number of studies	Estimate (95% CI)	Heterogeneity	
			*I*^2^	*p*-value
Male sex	5	1.38 (0.56, 2.20)	0.00%	0.81
Urban residence	3	1.01 (0.22, 1.81)	0.00%	0.76
Previous TB history	3	10.64 (−1.28, 22.56)	8.56%	0.46
DM duration more than 10 years compared with < 5 years	4	7.21 (−1.35, 15.76)	0.00%	0.96
Type 1 DM	4	2.70 (1.41, 3.99)	7.18%	0.42
BMI < 18.5 kg/m^2^	4	2.57 (−1.18, 6.32)	43.09%	0.17
HIV seropositive	4	1.42 (−0.19, 3.03)	0.00%	0.65
Family history of DM	3	1.42 (−0.19, 3.03)	0.00%	0.66
History of close contact with a known TB patient	5	5.73 (0.32, 11.13)	0.00%	0.76
Insulin medication	3	2.11 (0.71, 3.52)	0.00%	0.81
Smoking	4	5.63 (−2.79, 14.06)	2.08%	0.39
Alcohol	3	6.95 (0.79, 13.10)	0.00%	0.74
Poor glycemic control	4	1.51 (0.37, 2.66)	0.00%	0.93

DM: diabetes mellitus, TB: tuberculosis, BMI: body mass index, HIV: human immunodeficiency virus

**Figure 9:**
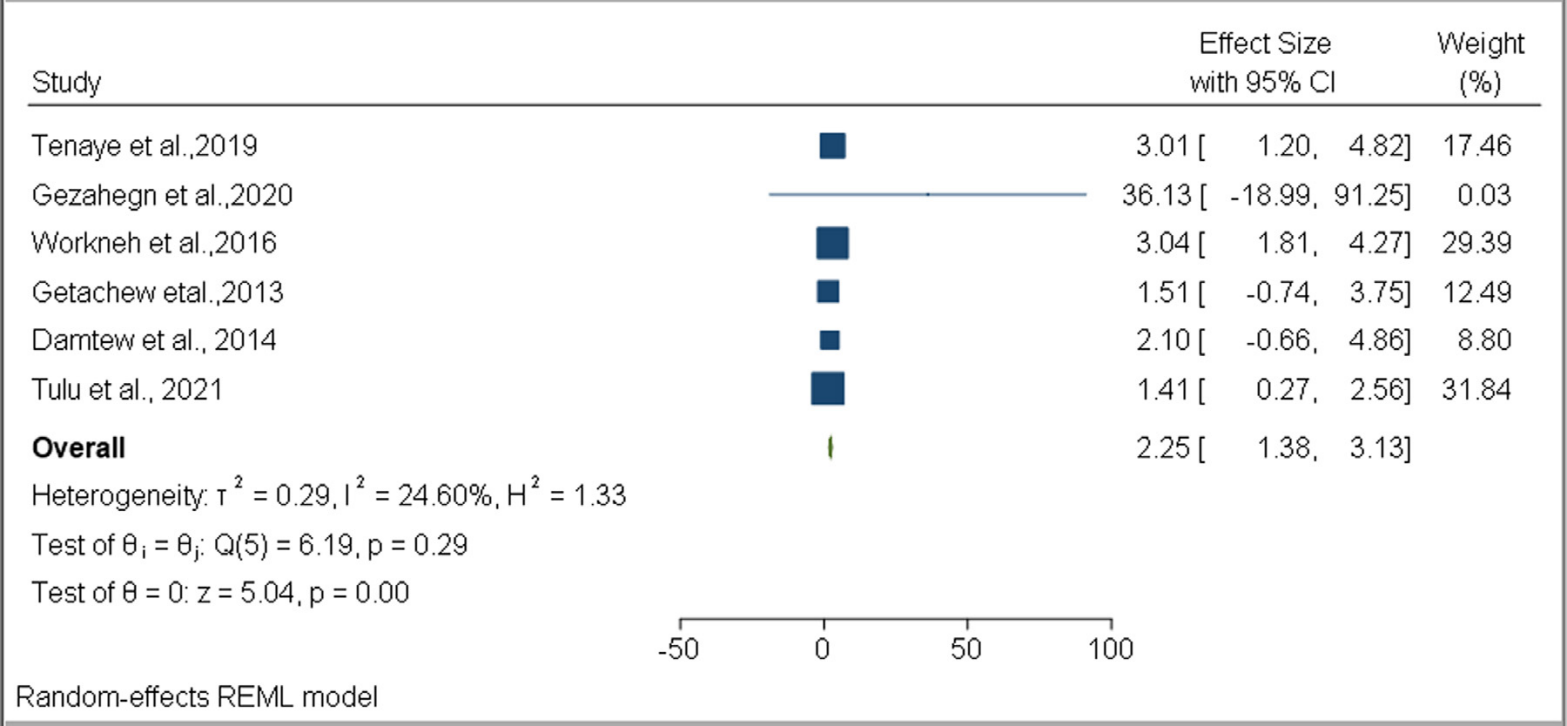
Forest plot for the association of older age with developing DM comorbidity among TB DM patients in Ethiopia

**Figure 10:**
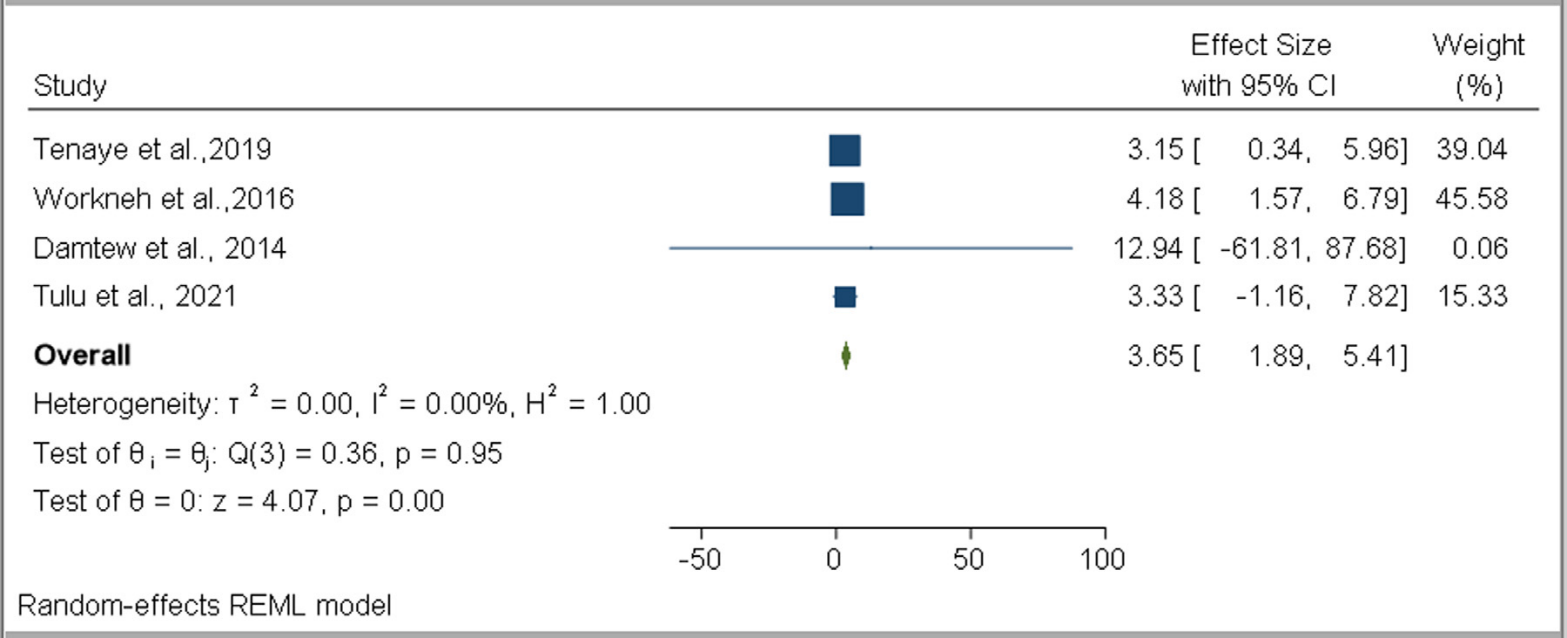
Forest plot for the association of family history of DM with developing DM-comorbidity among TB DM patients in Ethiopia

Among the variables assessed to determine the factors associated with DM co-occurrence among TB patients, two were found to have a statistically significant association: older age (OR 2.25, 95% CI 1.38–3.13, *I*^2^ = 24.60%) (Figure 9) and family history of DM (OR 3.65, 95% CI 1.89–5.41, *I*^2^ = 0.00%) (Figure 10). No statistically significant associations were found for female sex (OR 0.92, 95% CI 0.40–1.43, *I*^2^ = 79.83%), married (OR 1.39, 95% CI 0.60–2.18, *I*^2^ = 48.11%), urban residence (OR 0.68, 95% CI 0.38–0.97, *I*^2^ = 42.76%), smoking (OR 0.79, 95% CI 0.25–1.34, *I*^2^ = 0.00%), alcohol consumption (OR 0.82, 95% CI 0.49–1.14, *I*^2^ = 1.62%), khat consumption (OR 1.11, 95% CI 0.64–1.58, *I*^2^ = 30.99%), HIV (OR 0.80, 95% CI 0.14–1.47, *I*^2^ = 72.92%), overweight (OR 2.27, 95% CI 0.59–3.96, *I*^2^ = 0.00%), and smear-positive TB (OR 0.79, 95% CI 0.19–1.40, *I*^2^ = 56.02%) (Table 4 and Supplementary figure 3).

**Table 4:** Summary of pooled estimates of OR for associated factors for diabetes mellitus co-occurrence among tuberculosis patients in Ethiopia

Variable	Odds ratio			
	Number of studies	Estimate (95% CI)	Heterogeneity	
			*I*^2^	*p*-value
Female sex	7	0.92 (0.40, 1.43)	79.83%	< 0.001
Older age	6	2.25 (1.38, 3.13)	24.60%	0.29
Married	5	1.39 (0.60, 2.18)	48.11%	0.11
Family history of DM	4	3.65 (1.89, 5.41)	0.00%	0.95
BMI greater than 25	2	2.27 (0.59, 3.96)	0.00%	0.62
EPTB	4	0.77 (0.51, 1.03)	0.00%	0.63
Khat chewing	2	1.11 (0.64, 1.58)	30.99%	0.23
Smoking	4	0.79 (0.25, 1.34)	0.00%	0.69
Urban setting	7	0.68 (0.38, 0.97)	42.76%	0.19
HIV seropositive	4	0.80 (0.14, 1.47)	71.92%	0.01
Alcohol	3	0.82 (0.49, 1.14)	1.62%	0.39
Smear positive TB	5	0.79 (0.19, 1.40)	56.02%	0.08

DM: diabetes mellitus, TB: tuberculosis, EPTB: extrapulmonary tuberculosis, BMI: body mass index, HIV: human immunodeficiency virus.

## Discussion

This systematic review and meta-analysis study assessed the burden of TB and DM co-occurrences, and associated factors, in Ethiopia. Based on data extracted from the seven available studies, the pooled prevalence of TB among DM patients was estimated as 4.14% (95% CI 2.45–5.83%), while the pooled prevalence of DM among TB patients was estimated as 12.77% (95% CI 6.91–18.62%). Our study also revealed that type of DM was associated with developing TB among DM patients, while older TB patients (OR 2.25, 95% CI 1.38–3.13) and TB patients who had a family history of DM (OR 3.65, 95% CI 1.89–5.41) had a higher risk of developing DM compared with their counterparts.

Our study revealed that around 4.14% (4140 per 100 000 population) of DM patients in Ethiopia had TB. This was higher than the estimated national TB prevalence among the general population (140/100 000 population) (WHO, 2020). In a study carried out by Wagnew et al. (2018), using studies conducted in African and Asian countries, the pooled prevalence of TB among DM patients was estimated as 4.72%, with a pooled prevalence of 5.13% in Africa alone (Wagnew et al., 2018). Workneh et al., in their systematic review, revealed that the median overall global prevalence of TB among DM patients was 4.1% (Workneh et al., 2017). Another global pooled estimate revealed that DM patients had a two-to-four-fold increased risk of TB (Al-Rifai et al., 2017).

Our study also estimated the pooled prevalence of TB based on DM type. The findings revealed an 8.56% pooled TB prevalence among individuals who had type 1 DM, compared with 2.80% for individuals with type 2 DM. The pooled OR revealed that those individuals with type 1 DM had 2.70 times the odds for developing TB compared with type 2 DM patients. Likewise, a higher prevalence of TB among children and adolescents with type 1 DM was reported from South Africa (Webb et al., 2009), while a 10.0% prevalence of culture-positive PTB among type 1 DM patients was reported from India (Nair et al., 2016). The higher risk of TB in patients with type 1 DM might be due to the longer duration and difficulties in controlling hyperglycemia in this group, in addition to the lower body weights of young people who are mainly affected by type 1 DM.

Our study also assessed the prevalence of DM among TB patients. According to data collected from 3283 TB patients, 407 developed DM, giving a pooled prevalence estimate of 12.77%. This was lower than a global pooled estimate of 15.3% reported by Noubiap et al. (Noubiap et al., 2019). Likewise, in a study conducted by Workneh et al., the global pooled median prevalence of DM among TB patients was estimated as 16%. This might have been due to the high prevalence of DM among the general population in the countries included in the above studies. This was supported by a study conducted in South Asia, where the pooled prevalence of DM among TB patients was estimated as 21% (Gautam et al., 2021). However, in a study conducted by Alebel et al., the pooled estimate of DM prevalence among TB patients in sub-Saharan Africa was estimated as 9.0%, which is lower than that found by our study (Alebel et al., 2019).

Our study estimated the pooled prevalence of prediabetes among TB patients to be 24.19%. Although there have been no previously reported pooled estimates of the prevalence of prediabetes among TB patients, it has been commonly reported in individual studies. A comparable finding of 24.5% has been reported in India (Viswanathan et al., 2012). Higher prevalences have been reported in Vietnam (29%; Hoa et al., 2018) and Kenya (37.5%; Owiti et al., 2017), with a lower prevalence reported in Eritrea (10%; Araia et al., 2021). Generally, our study and previous studies conducted in different countries have revealed that TB patients are at high risk of developing DM, which necessities early DM detection among TB patients.

Of the 12 variables assessed using pooled OR estimates, two — older age and family DM history — were found to have a statistically significant association with DM in TB patients. Older TB patients had 2.25 times the odds for developing DM compared with their counterparts. A link between DM and older age was also reported in an earlier systematic review (Workneh et al., 2017). Likewise, a global meta-analysis study revealed that when the age of TB patients increased, the prevalence of DM also increased (Noubiap et al., 2019), while a nationwide cohort study in Portugual revealed that the odds for DM among TB patients increased by 4.7% per year of age (da Costa et al., 2016). The elderly are at risk of developing DM — mainly type 2 — due to decreased insulin secretion and impaired pancreatic islet functioning. A lack of physical activity and modern lifestyles, especially with regard to food choice, are the main factors resulting in obesity and consequent development of DM (Kirkman et al., 2012).

This study also revealed that TB patients with a family history of DM had 3.65 times the odds for developing DM co-occurrence compared with TB patients with no family DM history. This finding has been supported by a global systematic review (Workneh et al., 2017), while an individual study conducted in Tanzania revealed that TB patients who had a family history of DM had up to 17.5 times the odds for developing DM compared with their counterparts (Mabula et al., 2021). Other studies have also shown a family history of DM to be a major risk factor in developing DM in the general population (Zuo et al., 2014; Ismail et al., 2021).

Important limitations should be considered when interpreting the results of this study. First, the study was based on primary studies conducted only in the English language, which might have introduced bias. In addition, the majority of the studies were cross-sectional; this might have a limited the assessment of risk factors for co-occurrence. Furthermore, the small number of available primary studies might also have introduced bias. Finally, all the studies were hospital-based, which might not have reflected the nature of co-occurrences in the general population.

## Conclusion

Co-occurrence of TB and DM is a major public health problem in Ethiopia. The prevalence of TB among DM patients (4140/100 000 population) estimated in our study was far higher than the national TB prevalence (140/100 000 population) among the general population. Individuals with type 1 DM had a higher TB risk (8560/100 000 population) compared with those individuals with type 2 DM (2800/100 000 population). The estimated DM prevalence among TB patients (12.77%) was far beyond the national DM prevalence among adults (3.2%). This suggests the need for an integrative approach to decreasing the dual burden. Elderly TB patients with a family history of DM had a higher risk of developing DM; this needs to be considered during anti-TB treatment follow-up. Thus, active screening of DM patients for TB, and vice versa, is recommended.

## Author contributions

AA conceptualized, designed, and drafted the manuscript. AA, GD, and ZWB performed article searching, data extraction, and quality assessment. AA and ZWB conducted data analysis and wrote the manuscript. BG reviewed the final manuscript. All authors read, reviewed, and approved the final manuscript.

## Funding

The authors did not receive specific funding for this work.

## Availability of data and materials

All relevant data are available from the corresponding author upon request.

## Ethical approval and consent to participate

Not applicable

## Consent for publication

Not applicable

## Competing interests

The authors have declared that they do not have any competing interests.

## Appendix Supplementary materials

Image, application 1

Image, application 2
