# Supplementary material for: Co-occurrence of tuberculosis and diabetes mellitus, and associated risk factors, in Ethiopia: a systematic review and meta-analysis
Source: IJID Reg. 2021 Oct 20;1:82–91. doi: 10.1016/j.ijregi.2021.10.004 (PMC9216412; doi:10.1016/j.ijregi.2021.10.004)
Supplement: Supplementary file 1 [file mmc1.docx]

A B


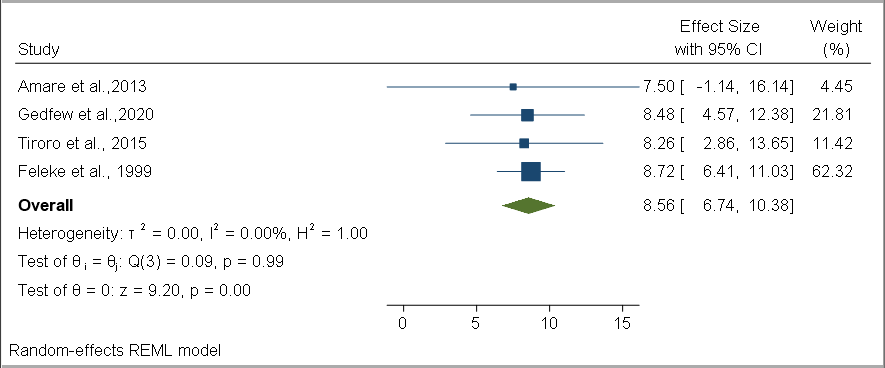

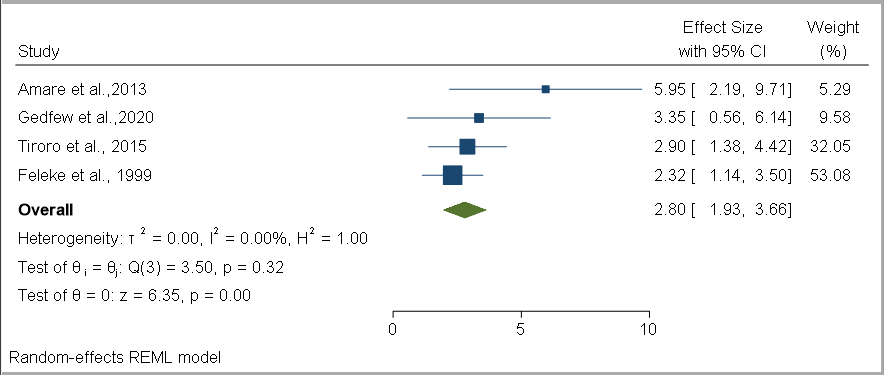


C D


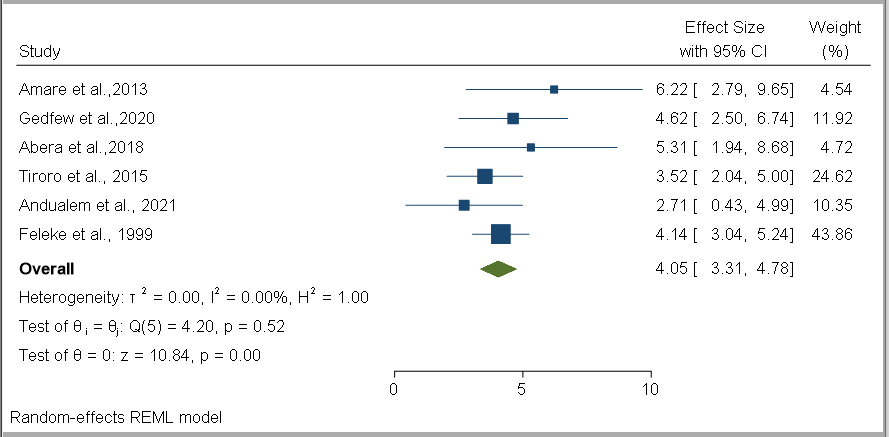

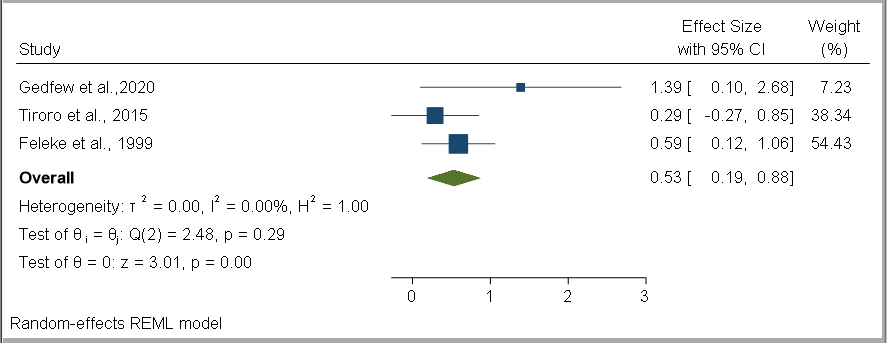


E F


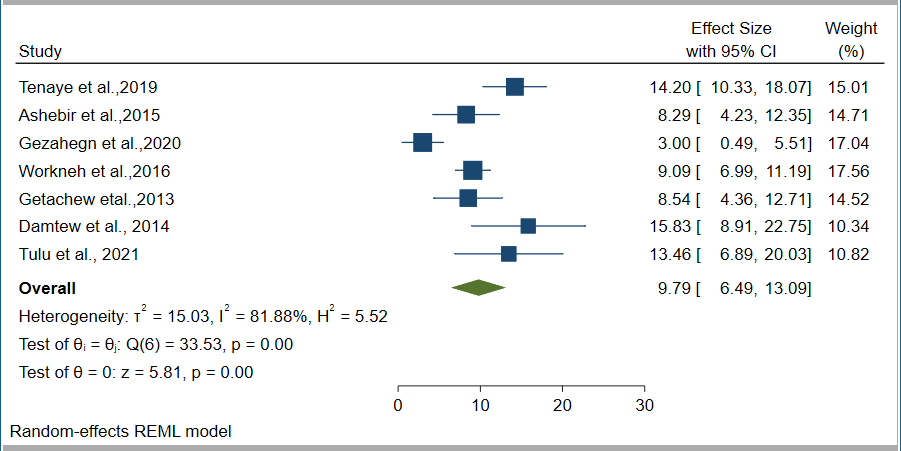

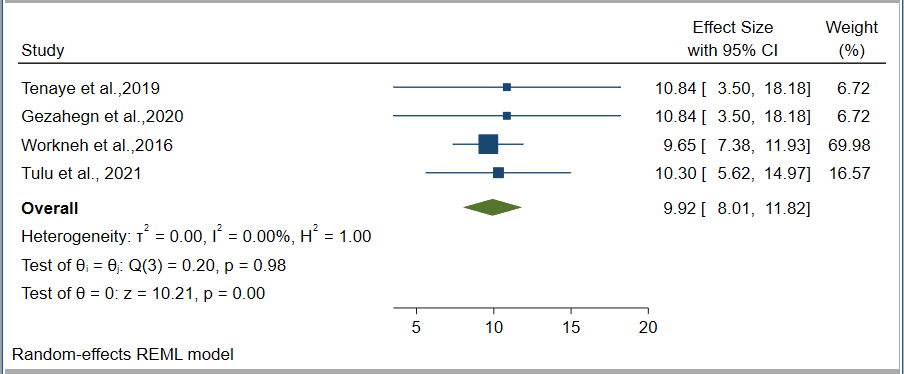


**Supplementary figure 1:** Forest plots for pooled prevalence estimates: A. TB among type 1 DM patients; B. TB among type 2 DM patients; C. PTB among DM patients; D. EPTB among DM patients; E. DM among PTB patients; F. DM among EPTB patients

A B C


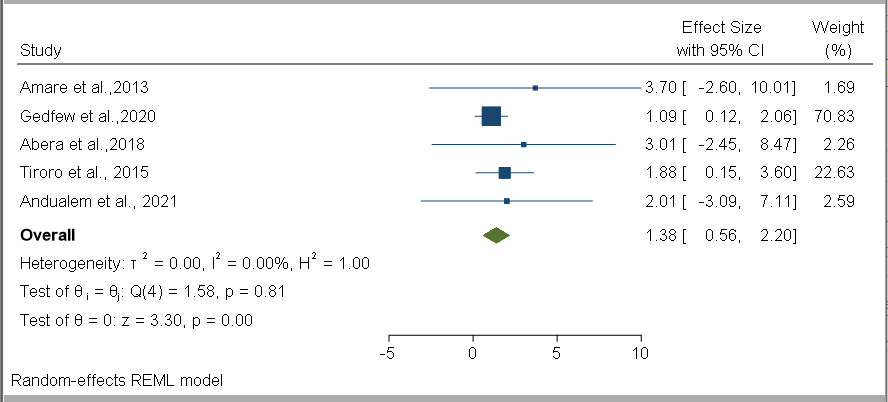

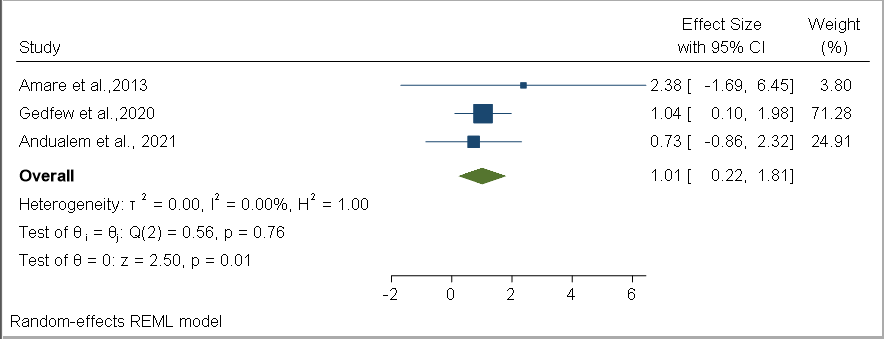

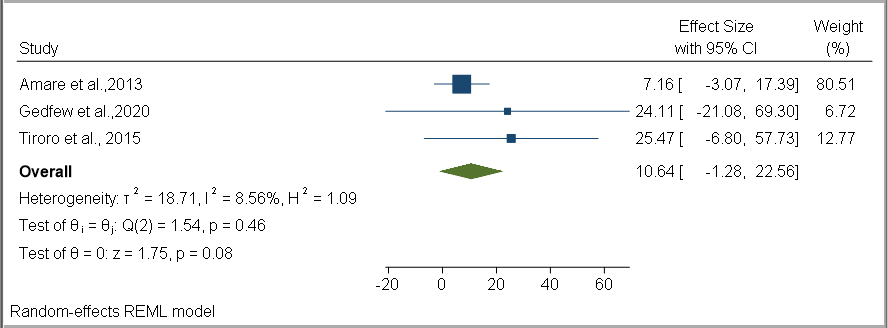


D E F


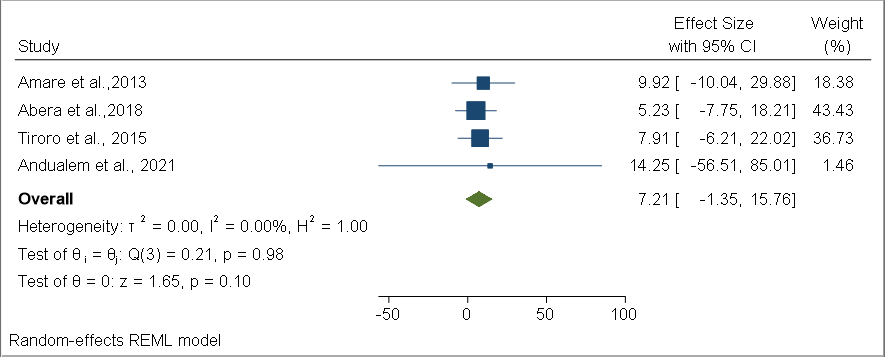

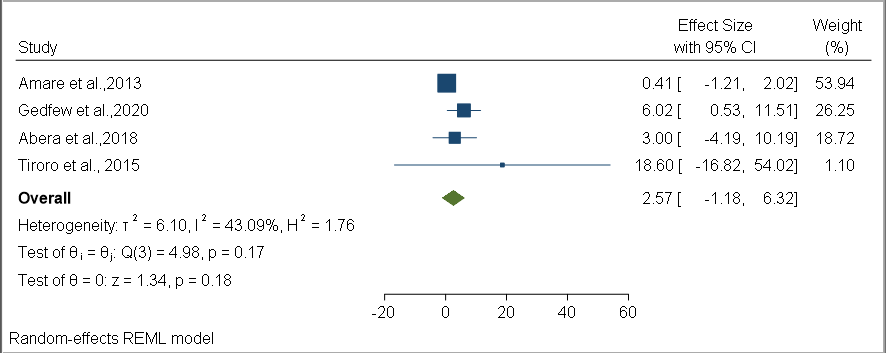

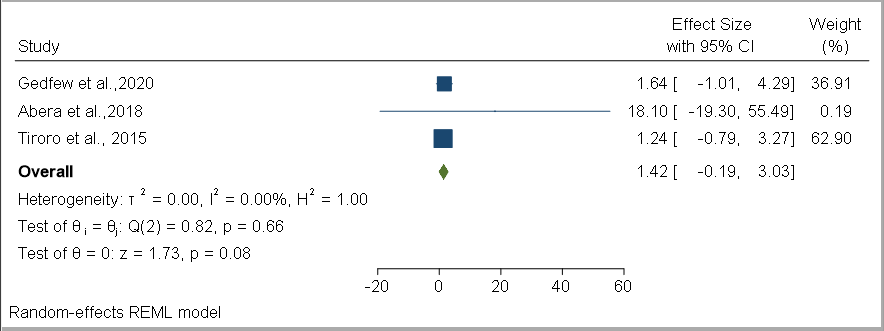


G H I


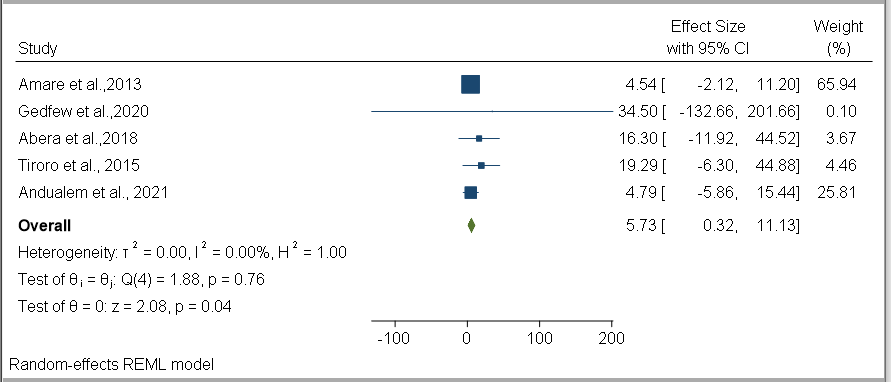

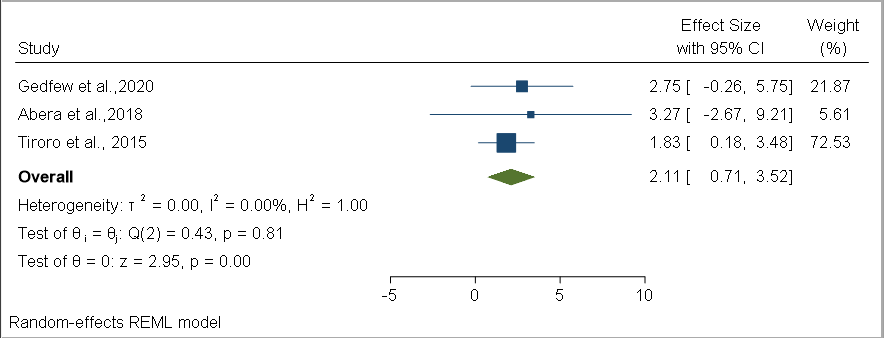

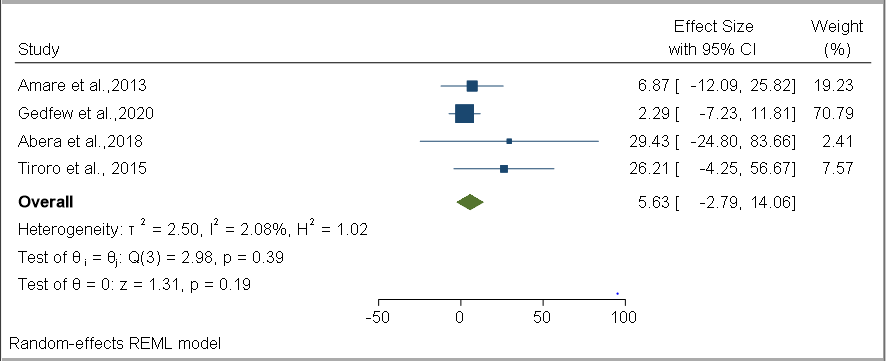


J K


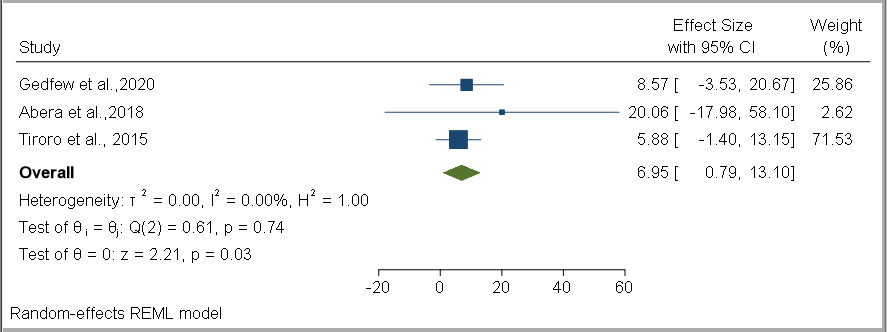

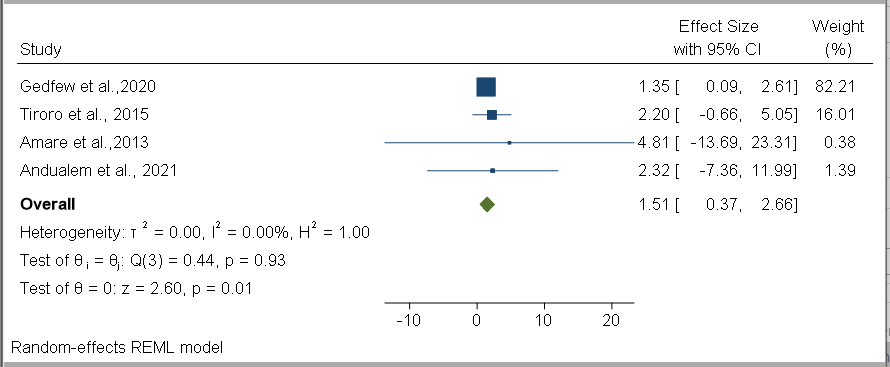


**Supplementary figure 2:** Forest plots for risk factors associated with TB infection among DM patients: A. male sex; B. urban residence; C. TB history; D. DM duration for > 10 years; E. BMI < 18.5–kg/m^2^; F. HIV seropositive; G. close contact with TB patient; H. insulin medication; I. smoking; J. alcohol consumption; K. poor glycemic control

A B C


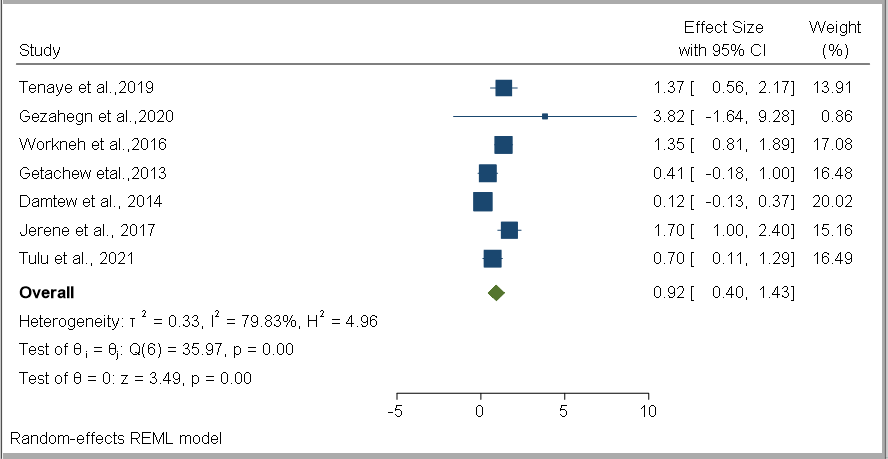

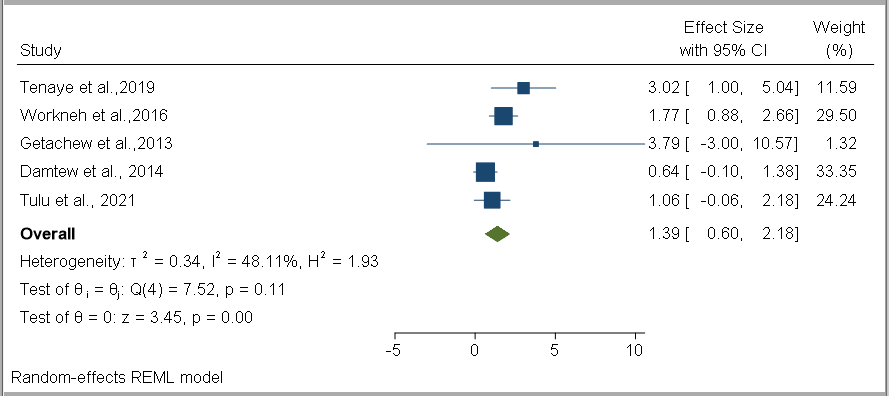

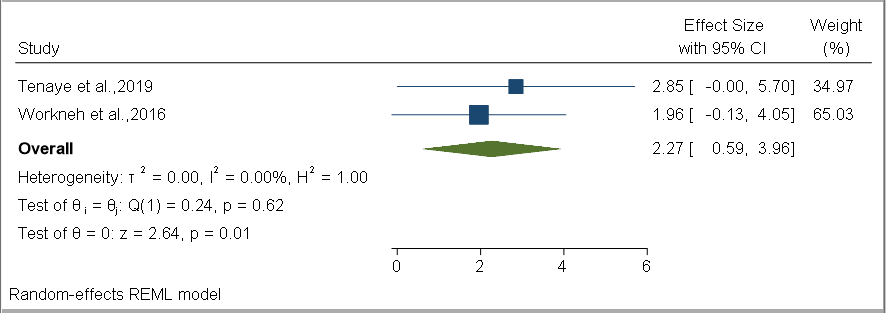


D E F


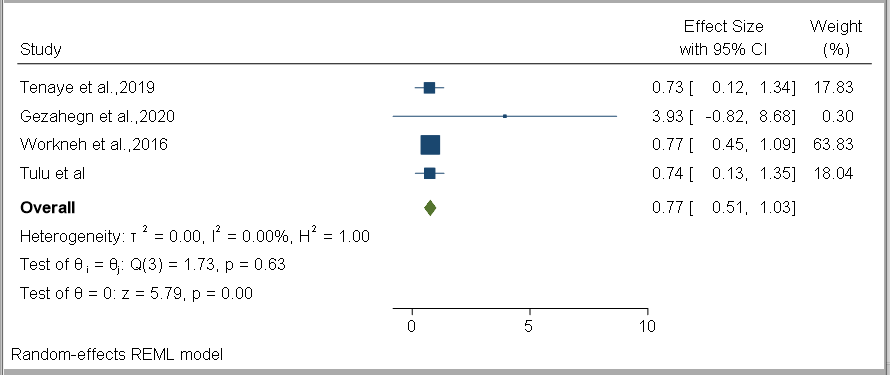

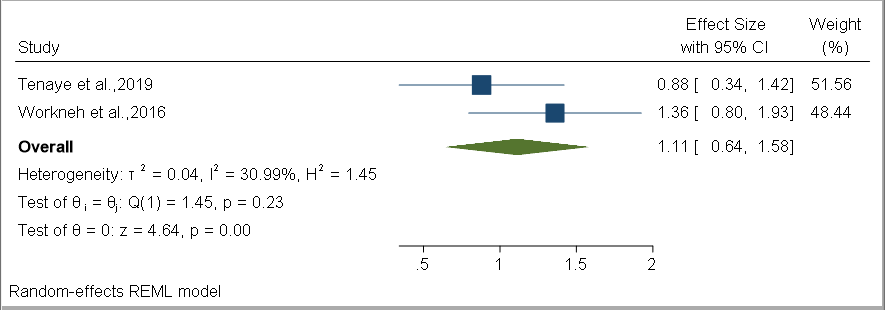

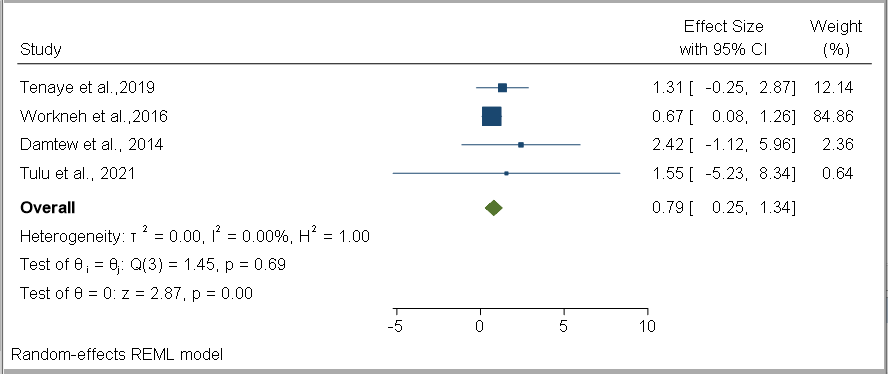


G H I


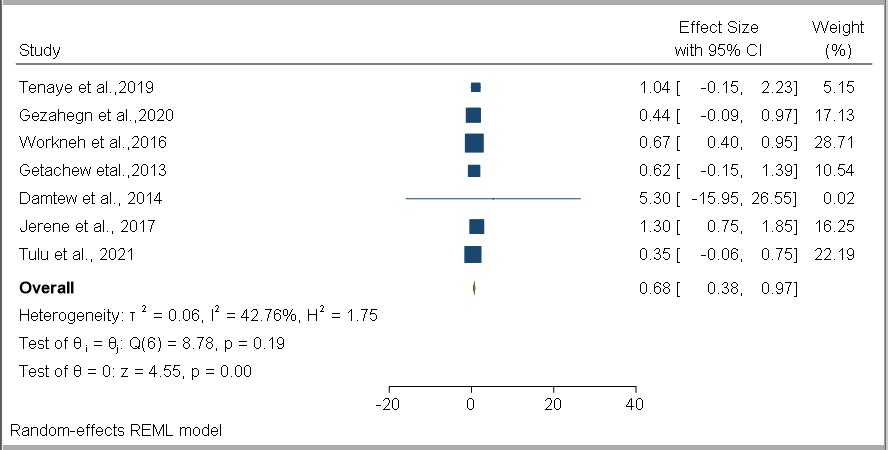

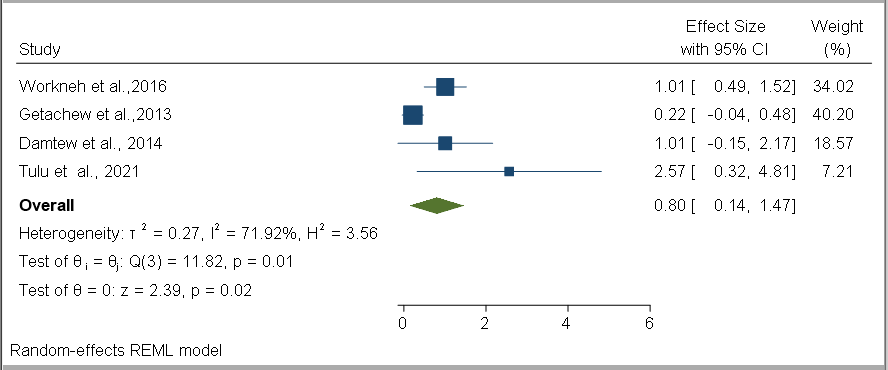

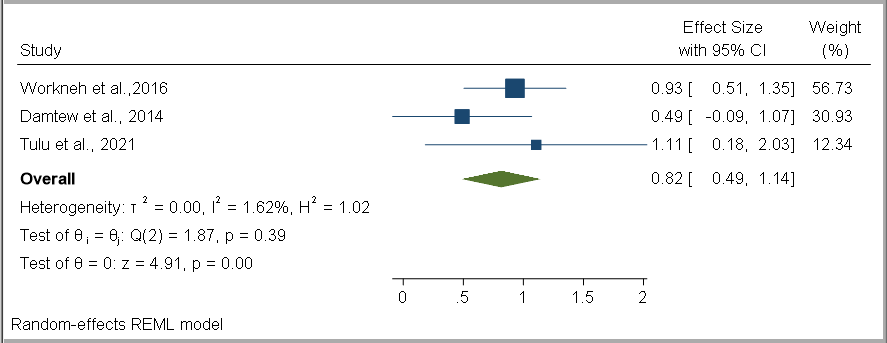


J


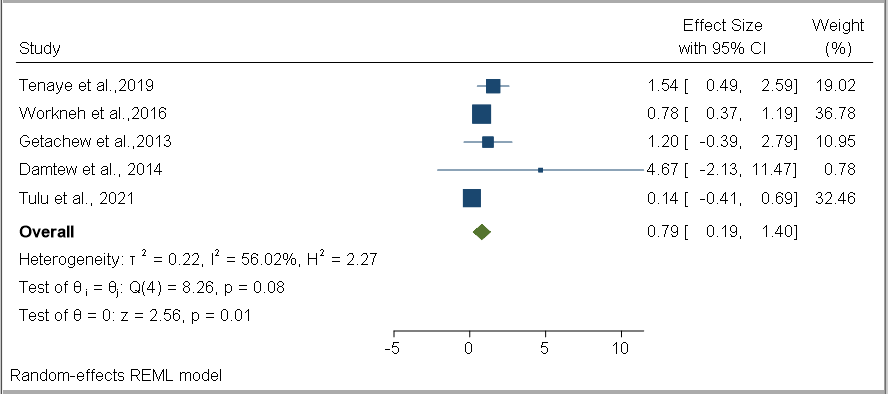


**Supplementary figure 2:** Forest plots for risk factors associated with DM co-occurrence among TB patients: A. female sex; B. married; C. overweight; D. EPTB; E. khat chewing; F. smoking; G. urban residence; H. HIV seropositive; I. alcohol consumption; J. smear positive TB
